# Triatomines in the city: A study of *Rhodnius neglectus* in Jaboticabal, São Paulo, Brazil, and its epidemiological implications

**DOI:** 10.1590/0037-8682-0133-2024

**Published:** 2024-11-15

**Authors:** Tiago Belintani, Jader de Oliveira, Vinícius Fernandes de Paiva, Jociel Klleyton Santos Santana, Isabella Maxwell Paulino Fernandes, Jonathan Silvestre Gomes, Estevam Guilherme Lux Hoppe, João Aristeu da Rosa

**Affiliations:** 1 Universidade Estadual Paulista, Faculdade de Ciências Farmacêuticas, Araraquara, SP, Brasil.; 2 Universidade Estadual Paulista, Instituto de Biociências, Rio Claro, SP, Brasil.; 3 Universidade de São Paulo, Faculdade de Saúde Pública, São Paulo, SP, Brasil.; 4 Universidade Federal do Acre, Programa de Pós-Graduação em Ciência, Inovação e Tecnologia para a Amazônia, Rio Branco, AC, Brasil.; 5 Universidade Estadual Paulista, Faculdade de Ciências Agrárias e Veterinárias, Jaboticabal, SP, Brasil.

**Keywords:** Chagas disease, Urbanization, Urban vectors, Vector control, Triatominae

## Abstract

**Background:**

Chagas disease, a significant global health concern rooted in social inequalities and political oversights, remains a challenging public health issue impacting millions worldwide. The increasing detection of triatomines, the vectors of Chagas disease, in urban areas complicates the situation.

**Methods:**

This study investigated the incidence of *Rhodnius neglectus* in the urban areas of Jaboticabal, São Paulo, Brazil, following several reports and previous collections of triatomines in the city. An educational approach was adopted, distributing informational materials and engaging the community through social networks to generate notifications that would enable the identification of triatomines. Specimens were collected using various methods, including passive surveillance actions, light traps, and active searches in palm trees.

**Results:**

*Rhodnius neglectus* was found in urban areas, invading homes in Jaboticabal, and was identified in palm trees. The educational approach led to the collection of 93 triatomines. Colonization was observed in a residence, with eggs, nymphs, and a concerning record of blood-feeding on a resident child. The houses where specimens were captured often had nearby palm trees with birds and nests, facilitating the passive transport of these insects and increasing the risk of invasion due to light attraction. No triatomines infected with *Trypanosoma cruzi* were identified.

**Conclusions:**

These findings emphasize the need for preventive measures to reduce the prevalence of *R. neglectus* in urban environments. The data elucidate the occurrence of *R. neglectus* in the city of Jaboticabal, associated with its potential behavioral adaptation in urban environments, underscoring the need for innovative control strategies.

## INTRODUCTION

Chagas disease is a global health problem deeply rooted in social inequalities and political negligence^1^ ^-^ ^3^. Its etiological agent, the hemoflagellate protozoan *Trypanosoma cruzi* (Chagas, 1909), is primarily transmitted through contact with the feces or urine of infected triatomine bugs^4^ ^-^ ^7^. Endemic to 21 Latin American countries, the disease has also been reported in other regions of the world^8^.

The interaction between triatomines, trypanosomatids, and humans in the Americas dates back to the pre-Columbian period, with evidence of *T. cruzi* infection dating back 9,000 years^9^ ^,^ ^10^. Despite this long history, it is only in recent decades that control initiatives have managed to reduce the incidence of this disease^11^. It is estimated that 6-7 million people are infected worldwide, with thousands living in high-risk areas^3^. Triatominae, a subfamily of Reduviidae^12^, encompasses 159 species^13^ ^-^ ^16^, most of which are naturally found in wild environments and exclusively participate in the natural infection cycle^5^; however, some species have adapted to urban settings^17^ ^,^ ^18^.

In Brazil, Chagas disease has spread alongside migration, particularly during the European colonization of the country's interior in recent centuries. Rapid environmental changes caused by deforestation and agricultural expansion have led to the adaptation of triatomines, vectors of the disease, to artificial environments, such as human dwellings, thereby increasing contact with humans and the risk of disease transmission^18^. 

An example of this dynamic is the colonization of urban areas in the Metropolitan District of Caracas, where occasional outbreaks of foodborne transmission occur^19^. Active transmission, although less significant, was also observed, mainly by species such as *Panstrongylus geniculatus* (Latreille, 1811), found in domestic areas. Other species collected from these areas included *Triatoma nigromaculata* (Stål 1859), *Triatoma maculata* (Erichson, 1848), *Rhodnius prolixus* Stål 1859, and *Panstrongylus rufotuberculatus* Stål 1859^19^. This study highlights the transmission interface between wild and urban environments and suggests that although direct transmission to humans is limited, the cumulative risk in a large population warrants further research and preventive measures^20^.

Comprehensive niche modeling and mapping of potential distributions of members of the *T. brasiliensis* complex revealed that *T. b. brasiliensis* Neiva, 1911 has the greatest potential to colonize new areas^21^. Although model projections do not anticipate many changes in the complex's distribution due to climate change, the epidemiological importance and dispersal abilities of the members of the complex are critical for understanding human exposure patterns^21^. This suggests that as habitable areas expand due to climate change, new infestations may occur, primarily from wild areas to urban environments^20^.

In São Paulo, Brazil, the primary vector for Chagas disease historically was *Triatoma infestans* (Klug, 1834), likely introduced in the 18th century. However, successful control measures targeting this species have led to the emergence of other relevant vectors, such as *Triatoma sordida* (Stål, 1859), *Panstrongylus tibiamaculatus* (Pinto, 1926), *Panstrongylus megistus* (Burmeister, 1835), and *Rhodnius neglectus* Lent, 1954^22^. The increasing detection of triatomines in urban areas, with an emphasis on secondary species, raises questions regarding the ability of these vectors to occupy ecological niches traditionally dominated by primary species^23^. This species replacement may indicate the greater ecological and adaptive plasticity of secondary species, allowing them to explore new habitats and food resources in urban environments^23^. Secondary species may exhibit different behavioral patterns, susceptibility to insecticides, and interactions with hosts compared to primary species, requiring specific management approaches^24^. 

Between 2013 and 2017, in the state of São Paulo-Brazil’s most populous and developed state (IBGE, 2023)-over 15,000 triatomine bugs were captured across 456 municipalities, encompassing 70% of the state’s territory^22^. The noteworthy species for entomological surveillance in the state include *P. megistus*, which has a high infection rate and the ability to colonize households; *T. sordida*, typically found near homes with lower *T. cruzi* infection rates; and *R. neglectus*, which is concentrated in the São Paulo Plateau, with a high capture rate in urban areas^22^.

*Rhodnius neglectus,* described by Herman Lent in 1954, is found in 14 Brazilian states and in the Federal District, where it harbors *T. cruzi* ^25^ ^-^ ^27^. This triatomine prefers to feed on birds, though it also feeds on mammals, including humans^28^. Despite their ability to fly, these insects are typically attracted to artificial light sources and move on foot in search of food^5^. Although the rate of *T. cruzi* infection is normally low in *R. neglectus* captured in artificial environment^22^ ^,^ ^29^, the presence of infection in such settings remains noteworthy. *R. neglectus* has already been found to have an infection rate of 15.9%^30^. 

The presence of triatomines in urban areas poses a significant challenge to Chagas disease control, requiring a comprehensive approach that integrates entomological surveillance with the One Health principles^31^ ^-^ ^33^. The adaptation of these vectors to urban environments, including domiciliation and blood-feeding behavior in humans, highlights the urgency of monitoring and control actions. Active surveillance-through searches, traps, and community engagement-is crucial for assessing population density, *T. cruzi* infection, and the presence of triatomines in urban areas, enabling the identification of at-risk areas and the implementation of targeted control measures^34^.

Integrating data on vector distribution, *T. cruzi* infection, and environmental factors influencing disease transmission is essential for developing more effective control strategies and promoting the health of affected communities^35^. In this context, the present study investigated the incidence of *R. neglectus* in the municipality of Jaboticabal, São Paulo, Brazil, including a description of its domiciliation and blood-feeding behavior in humans, with the aim of contributing to the understanding of Chagas disease dynamics in urban areas and supporting control actions^25^ ^,^ ^26^_._

## METHODS

### ● Geographical position and features of the study region 

**FIGURE 1:**
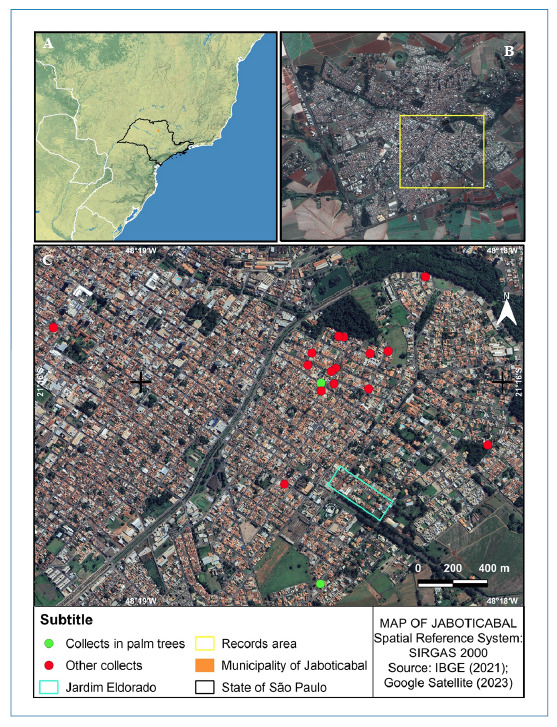
Map of the State of São Paulo **(A)** with emphasis on the municipality of Jaboticabal, highlighting the area of notifications and records of *R. neglectus* **(B)**. Collection points **(C)**.

 Jaboticabal is a municipality located in the State of São Paulo, covering a total area of 706,602 km^2^ (or 70,660 ha) (Figure 1). As of 2021, it sustains a population of 71,821 individuals, according to data from the Brazilian Institute of Geography and Statistics (IBGE)^36^. Situated within the metropolitan region of Ribeirão Preto, Jaboticabal has a tropical climate, characterized by mild temperatures and distinct wet and dry seasons^36^. This region is part of the Cerrado biome, renowned for its savanna-like landscape and rich biodiversity^37^. This environment is particularly valuable for studying *R. neglectus* in urban areas due to its ecological resemblance to the primary distribution areas of *R. neglectus* ^25^, making it an ideal model for research.

### ● Development of educational materials

To capture the triatomines, an awareness-raising strategy was implemented, involving the distribution of informational materials to residents of the study area, health professionals, and surveillance agents. The educational material, presented in the form of a banner, contained comprehensive information on Chagas disease, including epidemiological data, pathogenesis of the disease, clinical manifestations, guidelines for prevention and treatment, as well as the description of the vectors, their feeding habits, life cycle, and how to capture and store them. This banner was presented to community health and endemic agents, as well as to the general population through social media. Additionally, messaging applications were used to disseminate information and encourage community participation. The purpose of this material was not only to raise awareness but also to empower residents to identify potential triatomines and understand the disease cycle, fostering their active participation in surveillance efforts. The material was disseminated in health centers and through social media platforms.

### ● Strategy for Triatominae collection

After initially contacting the residents and obtaining preliminary information, the research team, supported by the Vector and Zoonoses Surveillance Department of the Municipality of Jaboticabal, proceeded to their homes to conduct active searches for triatomines. Light traps were installed in areas where no traces of the species were found. All information and reports provided by the residents were documented and stored. Homes where the specimens were found were inspected in detail, and residents were advised to contact local health authorities for a full risk assessment.

Specimens were collected using active methods, such as active searches and light traps, and traps based on the model proposed by Calor and Mariano^38^. Additionally, palm trees in the vicinity of residences were scrutinized using cranes, with specimens manually collected when necessary. The team systematically gathered essential data, including detailed photographic records and georeferenced locations, which provided valuable insights. Before installing traps in residences, property owners were diligently informed, and their consent was obtained through the completion of an Informed Consent Form (Supplementary material 1). The specimens captured were categorized by type of environment, stage of development, and physiological condition (e.g., regurgitated, alive, or dead). It is important to note that the local government did not directly participate in the study, however, one of the authors, responsible for vector and zoonoses surveillance in the municipality of Jaboticabal, provided support and authorization for the field activities. This study received no financial support from local government for the research^32^.

###  ● Analysis of natural infection and isolation of *T. cruzi* in triatomines 

 The investigation of natural infection by *T. cruzi* was carried out at the Parasitology Laboratory of the Universidade Estadual Paulista (UNESP), Faculdade de Ciências Farmacêuticas, Araraquara, SP, Brazil. The adopted technique involved examining the intestinal contents of the collected specimens under an optical microscope. The insect feces were accessed by abdominal compression, deposited on slides, diluted in phosphate-buffered saline, and covered with a coverslip for observation according to the methodology described by Ribeiro et al.^31^. 

### ● Triatominae identification

**FIGURE 2:**
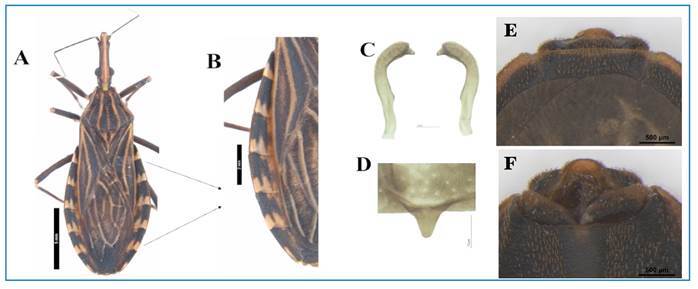
Female *Rhodnius neglectus* **(A)** Dorsal view. **(B)** Detail of the connexivum. **(C)** Male genitalia parameters. **(D)** Male pygophore median process. **(E)** Dorsal view of the female external genitalia. **(F)** Ventral view of the female external genitalia. These features contribute to the entomological identification of *R. neglectus*.

 After investigating natural infection by *T. cruzi,* live and dead specimens were preserved in absolute alcohol and stored at -80°C in the Laboratory of Virology at Universidade Estadual Paulista (UNESP), Faculdade de Ciências Agrárias e Veterinárias, Jaboticabal, SP. Later, they were transferred to the Parasitology Laboratory of the Universidade Estadual Paulista (UNESP), Faculdade de Ciências Farmacêuticas, Araraquara, SP, Brazil, where they were permanently stored in the laboratory's collection and identified using the key provided by Galvão^28^. To describe the morphology, the specimens were photographed using a Leica M205 stereo microscope and Leica Application Suite X (RRID:SCR_013673) image analysis system. Subsequently, image boards were created using image editing software to compare and describe the structures. For morphological studies, the pygophores were first removed from the abdomen using forceps and then cleaned in a 20% NaOH solution for 24 h. The dissected male and female genital structures were observed and photographed in glycerol using a Leica M205 stereoscopic microscope and Leica Application Suite X (RRID:SCR_013673), following the procedure described by Oliveira et al.^39^. The insects were examined dorsally, and the dissected male and female genitalia were evaluated. Observations and identification were performed as described by Oliveira et al.^40^ to confirm the species (Figure 2).

## RESULTS

In accordance with our objectives, we present the results of this study. The primary objective was to improve public awareness and education on triatomine recognition while investigating their distribution in urban areas with increasing reports of invasions. We implemented several strategies, including disseminating information through newsletters, conducting interactive lectures, displaying banners, and establishing communication channels via popular messaging apps and social networks in the region. These efforts have resulted in significant advancements in the collection and identification of *R. neglectus* specimens, thereby elucidating their prevalence in the urban environments of Jaboticabal.

Collections conducted across different neighborhoods of Jaboticabal between August 21, 2019, and September 6, 2023, revealed the presence of *R. neglectus* in urban environments. The captured specimens were categorized and summarized as shown in Table 1. The following methods were employed for collecting insects: Passive Surveillance Action (PSA) with 22 collections, Light Trap with 3 collections, and Active Search (AS) with 2 collections. PSA was the most frequently used method, followed by light traps and AS, with AS being employed in significant collections, such as on October 1, 2019, when 42 live specimens were captured through an active search in palm trees (Table 1).

**TABLE 1:** Information regarding address, date, location, ecotypes, physiological condition and quantity of triatomines collected in the Municipality of Jaboticabal, São Paulo.

Neighborhood	Collection date	Type of ecotope	Physiological Condition	Method of collection	number
Jardim Santa Rita	08/21/2019	intradomiciliary	Dead	PSA	**2**
Jardim Santa Rita	08/29/2019	peridomiciliary	Alive	light trap	2
Jardim Santa Rita	08/30/2019	intradomiciliary	Alive	PSA	**2**
Jardim Santa Rita	09/05/2019	intradomiciliary	Alive	PSA	**1**
Jardim Santa Rita	09/07/2019	intradomiciliary	Alive	PSA	**1**
Nova Jaboticabal	09/10/2019	intradomiciliary	Alive	PSA	**1**
Jardim Santa Rita	09/14/2019	peridomiciliary	Alive	light trap	1
Jardim Santa Rita	09/14/2019	peridomiciliary	Alive	light trap	1
Jardim Santa Rita	09/16/2019	intradomiciliary	Alive	PSA	**1**
Jardim Santa Rita	09/17/2019	intradomiciliary	Alive	PSA	**1**
Jardim Tangará	09/18/2019	peridomiciliary	Alive	PSA	**1**
Jardim São Marcos II	09/19/2019	intradomiciliary	Alive	PSA	**1**
Jardim Santa Rita	09/21/2019	intradomiciliary	Dead	PSA	**1**
Centro	09/21/2019	intradomiciliary	Dead	PSA	**1**
Jardim Santa Rita	09/26/2019	intradomiciliary	Alive	PSA	**1**
Nova Jaboticabal	09/26/2019	peridomiciliary	Dead	PSA	**1**
Jardim Santa Rita	09/28/2019	intradomiciliary	Alive	PSA	**1**
Nova Jaboticabal	09/30/2019	intradomiciliary	Alive	PSA	**1**
Colina Verde	09/30/2019	peridomiciliary	Alive	PSA	**1**
Jardim Santa Rita	09/30/2019	peridomiciliary	Alive	AS	16*
Jardim Santa Rita	10/01/2019	peridomiciliary	Alive	AS	42*
Jardim Tangará	10/09/2019	intradomiciliary	Alive	PSA	**1**
Jardim Santa Rita	10/09/2019	intradomiciliary	Dead	PSA	**1**
Jardim Santa Rita	10/09/2019	intradomiciliary	Dead	PSA	**1**
Nova Jaboticabal	10/10/2019	intradomiciliary	Dead	PSA	**1**
Jardim Santa Rita	10/14/2019	intradomiciliary	Alive	PSA	**2**
Jardim Santa Rita	10/15/2019	intradomiciliary	Dead	PSA	**1**
Jardim Santa Rita	10/18/2019	intradomiciliary	Dead	PSA	**1**
Jardim São Marcos	12/30/2019	intradomiciliary	Dead	PSA	**1**
Jardim Eldorado	09/06/2023	intradomiciliary	Alive	PSA	**4**
Total					**93**

*Collection carried out through active search in palm trees. **PSA:** passive surveillance action; **AS:** active search. Numbers in bold represent invasions.

The number of insects collected varied according to location and collection method. Collections were performed in both intradomiciliary and peridomiciliary ecotopes. The majority of *R. neglectus* specimens were found in intradomiciliary environments, indicating a substantial presence within residences (Table 1). A total of 93 specimens were collected from seven neighborhoods in Jaboticabal based on 24 notifications from the population, with the highest single collection yielding 42 specimens (Jardim Santa Rita, October 1, 2019, AS method). Most captured insects were alive, suggesting their adaptability to urban environments. In total, 21 collections were conducted in intradomiciliary settings, while 9 occurred in peridomiciliary settings.

During the study period, multiple collections of *R. neglectus* across different neighborhoods of Jaboticabal revealed varying indices of the incidence of these insects in urban environments. Jardim Santa Rita neighborhood had the highest number of collections, with a total of 15 events, representing a significant infestation focus. Significantly, collections conducted on September 30 and October 1, 2019, using the AS method on palm trees, resulted in the capture of 16 and 42 live insects, respectively, indicating a high population density.

The Nova Jaboticabal neighborhood has four collections distributed between intradomiciliary and peridomiciliary environments, with a predominance of live insects. In Jardim Tangará, two intra-domiciliary collections were conducted, resulting in the capture of live insects. Jardim São Marcos II and Jardim São Marcos were collection points, each contributing to one live insect capture event. In Centro, two intradomiciliary collections captured dead insects. Finally, in Colina Verde, a peridomiciliary collection resulted in the capture of one live insect, whereas the most recent collection in Jardim Eldorado on September 6, 2023, resulted in the capture of four live insects in an intradomiciliary environment. These data indicate variability in the distribution and density of *R. neglectus* across different neighborhoods of Jaboticabal, with a notable presence in Jardim Santa Rita and a lower but significant incidence in other neighborhoods. The concentration of collections and the number of specimens captured suggest the need for specific control measures for each neighborhood, with a particular focus on areas with a higher vector population density.

Despite informal records of triatomine sightings, information about their biology and ecotopes remains scarce, even in ecologically favorable contexts. The present study addresses this knowledge gap, with active capture proving to be the most effective method, resulting in the capture of 58 live specimens (Table 1). Moreover, most live specimens were found on palm trees near residential areas. The presence of palm trees and birds near houses inadvertently creates a favorable environment for the vector to settle inside, corroborating the results of previous studies on triatomines.

**FIGURE 3:**
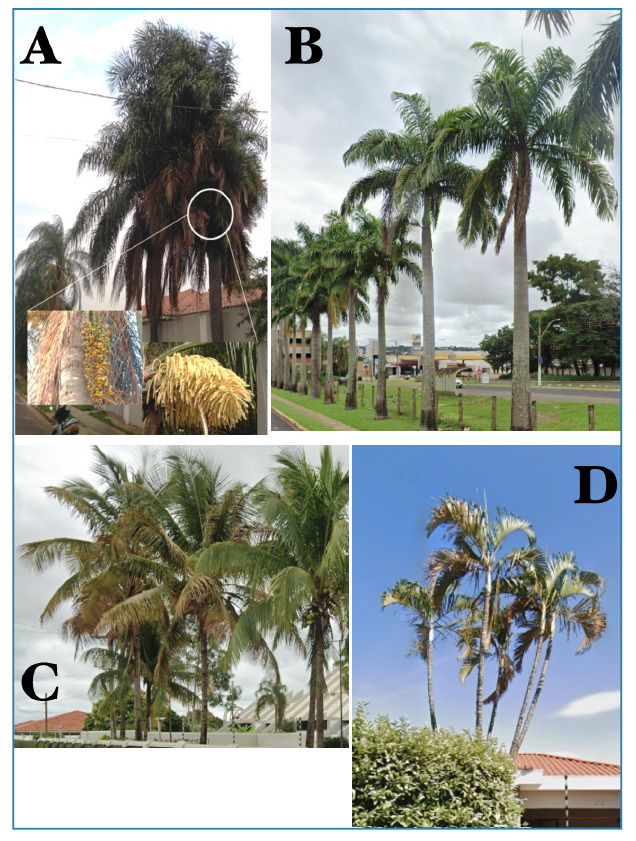
**A.** Palm tree where triatomine specimens were found. **B, C, and D.** Record of the high number of palm trees in the collection areas, highlighting the prevalence of these plants near residences.

Collections synchronized with palm tree pruning in Jardim Tangará (21°16'14.2"S, 48°18'36.2"W) and subsequent collection in Jardim Santa Rita (21°15'33.1"S, 48°18'35.4"W) revealed a notable prevalence of *R. neglectus* associated with palm trees, particularly the Jerivá palm (*Syagrus romanzoffiana*) and the Carnaúba palm (*Copernicia prunifera*), harboring a total of 16 specimens (Figure 3). These findings highlight the ecological importance of palm habitats for the abundance and distribution of triatomine vectors, particularly *R. neglectus*. Additionally, our records highlight the abundant presence of palm trees in landscaped corridors in the reported neighborhoods, emphasizing their potential roles as breeding sites and preservation areas for these vectors. For example, the palm trees identified were exotic to the Cerrado of São Paulo, primarily for landscaping purposes.

**FIGURE 4:**
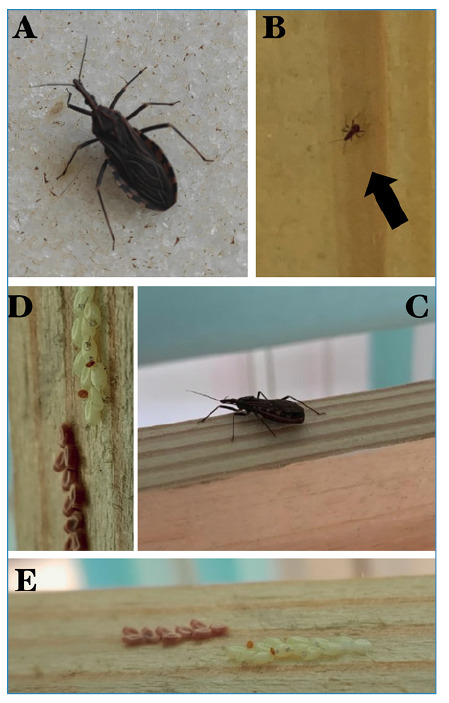
Record of the domiciliation of *R. neglectus* in a residence located in a middle-class neighborhood in Jaboticabal. **(A)** and **(C)** depict adults; **(B)** shows a nymph of *R. neglectus*; **(D)** and **(E)** show eggs and eggshells adhered to the bed.

**FIGURE 5:**
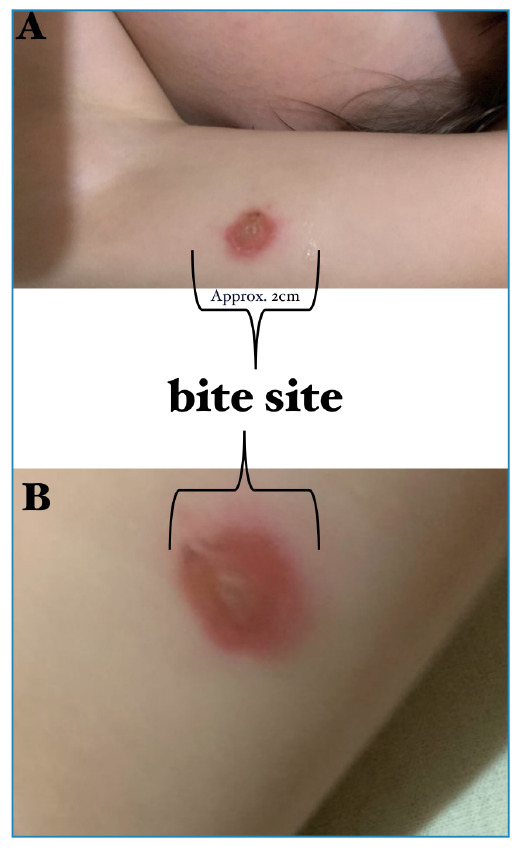
Record of a child residing in the house where *R. neglectus* is domiciled and had a blood meal. **(A)** General view of the bite region and the inflammatory process in the skin. **(B)** Highlight of the bite region.

In the Jardim Eldorado neighborhood in September 2023. During a home visit, an adult female triatomine was found in the bedroom of the youngest child along with fertile eggs and nymphs (Figure 4). This discovery prompted medical follow-up of the family, and subsequent tests did not indicate infection (Figure 5). 

The investigation of natural infection by *T. cruzi* was carried out at the Parasitology Laboratory of the Universidade Estadual Paulista (UNESP), Faculdade de Ciências Farmacêuticas, Araraquara, SP, Brazil. The intestinal contents of the insects were examined under an optical microscope. Insect feces were observed, and no protozoan forms were found.

Although we did not directly test the impact of our communication strategies, we observed a substantial improvement in the rate of positive notifications. This was likely due to the increased ability of the population to identify triatomines, their eggs, or traces, such as feces, which, in turn, allowed for faster confirmation of reports through collection and identification. This finding highlights the crucial role of community awareness in effective vector surveillance and control. Active community engagement further fosters a collaborative approach to disease prevention and control, instilling a sense of shared responsibility among residents. By fostering a culture of vigilance and encouraging prompt reporting of potential vector sightings, community members have become key partners in ongoing efforts to mitigate vector-borne diseases.

## DISCUSSION

Vector-borne diseases, including Chagas disease, pose significant challenges to public health worldwide, affecting millions of people across various regions. Despite their widespread prevalence, a substantial gap remains in understanding the key aspects of these diseases, such as the transmission dynamics of understudied species, vectorial capacity, and the role of coinfections, particularly in urban areas^30^. This scoping study aimed to address these gaps by providing effective communication channels and assessing the incidence of *R. neglectus* infections in Jaboticabal. 

The study assessed the regional importance of disseminating information to health care professionals, surveillance services, and the general public. The aim was to provide a better understanding of the incidence and distribution of vectors of neglected diseases, along with their associated health risks^41^. It is widely recognized that education plays a crucial role in combating neglected diseases; however, the public's awareness and involvement in entomological control and surveillance efforts remain variable. Studies underscore how gaps in public knowledge can significantly hinder the effectiveness of control strategies^42^ ^-^ ^44^.

Recent studies have shown that current technological resources, such as apps and the Internet, play crucial roles in advancing vector control strategies. For instance, the VetorDex application suite (https://play.google.com/store/apps/details?id=vetordex.com&hl=pt_BR) exemplifies innovative approaches aligned with current vector control objectives. TriatoDex^45^, a part of this suite, provides specialized tools for identifying various triatomine species through interactive features and detailed imagery to simplify the identification process and empower users to make informed decisions. These integrated technological solutions in public health initiatives can significantly enhance the precision and efficiency of vector surveillance and control, thereby strengthening disease prevention strategies.

*Rhodnius* spp., known for their preference for palm trees, establish close links with these trees and use them as shelters and for access to food sources^5^ ^,^ ^28^. While typically found in the nests of wild birds and palm trees, especially in the Cerrado biome^5^, *R. neglectus* is becoming increasingly common in urban areas^17^ ^,^ ^25^ ^,^ ^26^. Triatomines feed on various sources, including birds, amphibians, mammals, and reptiles^46^; however, birds have not been previously considered hosts of *T. cruzi* ^47^ ^-^ ^49^. Recent studies have challenged this perspective, suggesting that birds may serve as reservoirs of this parasite. For the first time, researchers have documented the presence of protozoan forms in wild birds, as reported by^50^. Protozoa found in wild birds, as documented by Martínez-Hernández et al.^51^, are particularly relevant in this context because the genus *Rhodnius* is closely related to birds. These birds not only provide food for insect vectors but also facilitate the passive transportation of these insects into homes. 

The close association between *Rhodnius* spp. and birds underscores the potential role of avian species in the transmission dynamics of *T. cruzi*. This ecological relationship highlights the need for comprehensive surveillance and control measures targeting both vector habitats and potential reservoirs to effectively mitigate the risk of Chagas disease transmission in both wild and urban environments. Understanding and addressing these complexities are crucial for developing integrated strategies to manage and reduce the burden of this neglected tropical disease.

Observations by Diotaiuti and Dias^52^ suggest that population density in the natural habitat of *R. neglectus* may correlate with food availability and the presence of predators. Despite a 15.9% infection rate of *T. cruzi*, primarily from marsupials, indicating *R. neglectus* is a significant sylvatic vector, this study found no evidence supporting *R. neglectus* as a direct transmitter of *T. cruzi* to humans in this area. These insights underscore the need for integrated surveillance and control strategies targeting both vector habitats and potential reservoirs to effectively manage Chagas disease transmission across diverse environments.

In urban environments, where palm trees and bromeliads are often used for landscaping, these plants play a crucial role in maintaining the enzootic cycle of *T. cruzi* by providing shelter and food for triatomines, mainly *Rhodnius* species^53^. This study identified colonies of *R. neglectus* on palm trees, with 42 specimens found in *Syagrus romanzoffiana* (Jerivá palm) and 16 specimens from *Copernicia prunifera* (Carnaúba palm). Effective management of landscaped palm trees and continuous monitoring are essential to mitigate the risk of vector-borne diseases. The regular inspection and maintenance of these plants can aid in the early detection and control of *Rhodnius* populations, thereby reducing their potential for disease transmission. 

Recently, Carbajal-del-Fuente et al.^18^ highlighted a significant increase in records of vectors in urban homes over the last three decades. This infestation covers cities of various sizes, from small towns to megalopolises, across the Americas, from Argentina to the United States^18^. In São Paulo state, some municipalities showed an increase in the capture of triatomines, such as the study in Araçatuba, municipality of São Paulo, in 2009, which collected 81 specimens of *R. neglectus* from human dwellings, with 2.7% of the samples indicating the presence of human blood, suggesting the possibility of transmission of the disease^26^. In addition, triatomine specimens were collected in the capital of São Paulo, the most developed region in Brazil^31^ ^,^ ^32^. 

This increase in the detection of triatomines in urban areas, including secondary species such as *R. neglectus*, raises concerns about the adaptation of these vectors to new environments and the potential expansion of their geographical distribution^18^. Global warming, with rising temperatures and changing rainfall patterns, may contribute to this expansion, creating more favorable conditions for the survival and reproduction of triatomines in previously inhospitable regions. 

The integration of technological innovation into health control programs, along with active community involvement and interdisciplinary collaboration, has facilitated the successful implementation of these strategies. This provides tangible benefits to the community and empowers individuals to report the presence of kissing bugs in their homes. The results underscore the crucial role of information channels, such as social networks and accessible messaging apps, in health-related contexts and Chagas disease control, even under less representative conditions. This successful approach has been replicated in other regions such as Guatemala, where a similar strategy yielded significant results in an area exposed to vectors such as *Triatoma dimidiata* Latreille, 1811^41^.

Although triatomines are typically found in socially disadvantaged areas, our results indicate a different trend^41^. The collections were conducted in socially developed regions, which can be characterized as middle-to high-class neighborhoods. This finding is consistent with the results of studies involving the same species in four municipalities in São Paulo^22^. Based on the available information, it is possible to speculate that there was a shift in the behavior of the kissing bugs. These findings indicate a worrisome trend of increasing triatomine presence in urban areas, even in developed regions, underscoring the importance of stringent vector control in preventing Chagas disease. This adaptation process is heavily influenced by human interactions and pressures, necessitating the adaptation and strengthening of vector control programs to address the shift from rural to urban environments. 

The presence of vectors in urban areas poses additional challenges for Chagas disease prevention and control, highlighting the importance of ongoing surveillance and intervention. Considering these challenges, the integration of community awareness and educational initiatives with systematic specimen collection and surveillance is imperative. By empowering communities with knowledge about triatomine recognition and control measures, coupled with regular specimen collection and reporting, we can enhance early detection and response efforts, ultimately mitigating the spread of Chagas disease in urban settings.

CONCLUSION

In this study, which addressed the presence of *R. neglectus* in a middle-class area of Jaboticabal, São Paulo, we highlight the importance of disseminating information among health professionals, surveillance services, and the general community was to understand the distribution of the vectors of neglected diseases and their associated risks. Raising public awareness played a fundamental role, using communication channels, such as social networks and messaging applications, which allowed for a better understanding of the presence of vectors in urban areas. The results also showed changes in the behavior of *R. neglectus* as well as its association with palm trees and birds. Consequently, it is necessary to consider these factors when developing strategies for Chagas disease control and prevention. The findings of this study underscore the need for proactive measures to address vector-borne diseases and emphasize the importance of community involvement in surveillance and control efforts. By leveraging existing communication platforms and fostering collaboration among various stakeholders, including health authorities, researchers, and residents, it is possible to enhance vector surveillance and mitigate the risk of disease transmission. However, it is important to note that, although occasional notifications from the population living in areas infested by triatomines are valuable for surveillance, they are not sufficient for effective vector control. It is essential that notifications are followed by active search actions, chemical treatment, and environmental management, in addition to continuous educational campaigns, to ensure community participation and reduce the risk of Chagas disease transmission. Continued research and vigilance are essential to monitor vector populations, identify emerging threats, and implement targeted interventions to protect public health. Through concerted efforts and community engagement, we can work towards effectively combating vector-borne diseases and safeguarding the well-being of populations in urban areas.
