# Supplementary material for: Triatomines in the city: A study of Rhodnius neglectus in Jaboticabal, São Paulo, Brazil, and its epidemiological implications
Source: Rev Soc Bras Med Trop. 2024 Nov 15;57:e00422-2024. doi: 10.1590/0037-8682-0133-2024 (PMC11654749; doi:10.1590/0037-8682-0133-2024)
Supplement: Supplementary file 1 [file 1678-9849-rsbmt-57-e00422-2024-supp1.pdf]

Supplementary material 1. Informed Consent Form Applied to adults and responsible for children (english language version). This consent form is in accordance with the Guidelines of Resolution No. 1 of the National Health Council, dated June 13, 1988, republished in the D.O.U. on January 5, 1989, and adapted from the University of Maryland - USA model.

INFORMED CONSENT FOR PARTICIPATION IN RESEARCH

Institution: São Paulo State University, Jaboticabal Campus, Faculty of Agricultural and Veterinary Sciences

COLLECTION AND IDENTIFICATION OF TRIATOMINES (HEMIPTERA: REDUVIIDAE: TRIATOMINAE) IN THE JABOTICABAL REGION, SÃO PAULO

VOLUNTEER INFORMATION

You, ____, are being invited to participate as a volunteer in a research project. You have the right to be informed about the procedures that will be carried out during your participation in this study. RESPONSIBLE RESEARCHERS: Dr. Estevam Guilherme Lux Hoppe – Veterinary Medical Doctor, Laboratory of Parasitic Diseases, São Paulo State University, Jaboticabal Campus, Faculty of Agricultural and Veterinary Sciences.

Isabella Maxwell Paulino Fernandes – Resident Veterinary Medical Doctor in the Veterinary Medicine and Health Residency Program, São Paulo State University, Jaboticabal Campus, Faculty of Agricultural and Veterinary Sciences.

As a volunteer, you are being asked to participate in a scientific project conducted by São Paulo State University, Jaboticabal Campus, Faculty of Agricultural and Veterinary Sciences. The project aims to collect triatomines (Hemiptera: Reduviidae: Triatominae) in intradomiciliary environments in the Jaboticabal region, São Paulo. You have the right to refuse to participate in this research without facing any embarrassment or penalties from the institution. Researchers are obligated not to disclose your identity in any publication resulting from this study. Before signing this form, you should fully inform yourself about it and do not hesitate to ask questions about any aspect you consider necessary to clarify. It is important to be aware of the following

information: a) Purpose of the research: the collection of triatomines (Hemiptera: Reduviidae: Triatominae) in intradomiciliary environments in the Jaboticabal region, São Paulo. b) Benefits: gaining knowledge on the subject and the development of educational materials that will be later provided to the municipal health service to inform the public about the subject under investigation.

Declaration

I hereby declare that I am aware of the full content of the Informed Consent Form for participation in the project 'Collection and Identification of Triatomines (Hemiptera: Reduviidae: Triatominae) in the Jaboticabal region, São Paulo,' and I have decided to participate in the proposed research after asking questions and receiving satisfactory answers.

Date: __________________,202___

Resident subscription: ______________________________
